# Mitochondrial Genome Variations in Advanced Stage Endometriosis: A Study in South Indian Population

**DOI:** 10.1371/journal.pone.0040668

**Published:** 2012-07-17

**Authors:** Suresh Govatati, Nageswara Rao Tipirisetti, Shyam Perugu, Vijaya Lakshmi Kodati, Mamata Deenadayal, Vishnupriya Satti, Manjula Bhanoori, S. Shivaji

**Affiliations:** 1 Department of Biochemistry, Osmania University, Hyderabad, Andhra Pradesh, India; 2 Department of Genetics, Osmania University, Hyderabad, Andhra Pradesh, India; 3 Vasavi Medical and Research Centre, Hyderabad, Andhra Pradesh, India; 4 Infertility Institute and Research Centre, Secundrabad, Andhra Pradesh, India; 5 Centre for Cellular and Molecular Biology, Hyderabad, Andhra Pradesh, India; Florida International University, United States of America

## Abstract

**Background:**

Endometriosis is a chronic gynecological benign disease that shares several features similar to malignancy. Mitochondrial DNA (mtDNA) mutations have been reported in all most all types of tumors. However, it is not known as to whether mtDNA mutations are associated with endometriosis.

**Methodology:**

We sequenced the entire mitochondrial genome of analogous ectopic and eutopic endometrial tissues along with blood samples from 32 advanced stage endometriosis patients to analyze the role of somatic and germ-line mtDNA variations in pathogenesis of endometriosis. All ectopic tissues were screened for tumor-specific mtDNA deletions and microsatellite instability (MSI). We also performed mtDNA haplogrouping in 128 patients and 90 controls to identify its possible association with endometriosis risk.

**Principal Findings:**

We identified 51 somatic (novel: 31; reported: 20) and 583 germ-line mtDNA variations (novel: 53; reported: 530) in endometriosis patients. The A13603G, a novel missense mutation which leads to a substitution from serine to glycine at the codon 423 of ND5 gene showed 100% incidence in ectopic tissues. Interestingly, eutopic endometrium and peripheral leukocytes of all the patients showed heteroplasmy (A/G; 40–80%) at this locus, while their ectopic endometrium showed homoplasmic mutant allele (G/G). Superimposition of native and mutant structures of ND5 generated by homology modeling revealed no structural differences. Tumor-specific deletions and MSI were not observed in any of the ectopic tissues. Haplogrouping analysis showed a significant association between haplogroup M5 and endometriosis risk (*P*: 0.00069) after bonferroni correction.

**Conclusions:**

Our findings substantiate the rationale for exploring the mitochondrial genome as a biomarker for the diagnosis of endometriosis.

## Introduction

Endometriosis is a chronic gynecological disorder that occurs in 10% of women of reproductive age and in up to 50% of women with infertility [1]. It is characterized by the presence of endometrium like tissue outside the uterine cavity. Endometriosis is a benign disease but shares some features with cancer [2]. Several mechanisms have been proposed to explain the development of endometriosis [3], but the etiology and pathogenesis remain unclear. Previously we demonstrated the correlation between single nucleotide polymorphisms (SNPs) of various candidate genes and endometriosis in Indian population [4]–[9]. The emerging evidence strongly suggests that the disease has a polygenic and multifactorial basis [10]. Factors that induce alterations in the morphology of the peritoneal mesothelium have been reported in menstrual fluid of endometriosis patients [11]. Oxidative stress may be responsible for local destruction of the peritoneal mesothelium followed by the development of adhesion sites for ectopic endometrial cells [12]. Elevated oxidative stress markers have been found in the serum and peritoneal fluid of endometriosis patients [13]–[14]. Increased reactive oxygen species (ROS) production, higher endogenous oxidative stress and alterations in ROS detoxification pathways have been reported in endometriotic cells from patients [15]. Mitochondria are a major source for ROS generation. Mutations in mitochondrial DNA (mtDNA) have been found to increase ROS production and enhance metastatic potential of tumors [16].

Mitochondria are semiautonomous organelles and have a variety of important roles to play, including energy metabolism, generation of ROS, aging, and regulation of apoptosis [17]. Hence mitochondria may serve as the switching point between cell death and abnormal cell growth. Human mtDNA (16.569 kb) encodes 13 subunits of the electron transport chain (ETC), as well as a distinct set of rRNAs and tRNAs [18]. It exhibits higher mutation rate than nuclear DNA and is more susceptible to oxidative damage due to a lack of protective histone proteins, limited DNA repair mechanisms and a high rate of ROS generation [19]. During the course of evolution, several mutations have accumulated in the mtDNA that allows human populations to be categorized in to various discrete, region specific, mitochondrial clades or haplogroups. Recent investigations showed an association of mtDNA haplogroups with various diseases including cancer [20]–[21]. However, studies investigating the role of mitochondrial haplogroups in pathogenesis of endometriosis are not well explored and studied.

The mtDNA mutations could arise either in the germ-line and predispose cancer or in somatic tissues and participate in the tumor progression process. Both types of mutations have been reported in various types of tumors [22]. Although the molecular genetics of endometriosis has been in the focus of many research laboratories for a long period of time, relevant prognostic and diagnostic markers are still missing. At the same time mtDNA mutations have been reported in various types of tumors during the last two decades [22]. It is therefore very likely that the mitochondrial genotype is one of the tumor susceptibility factors. We propose that mtDNA mutations and/or haplogroups might be anticipated in the initiation or progression of endometriosis. To the best of our knowledge, no screening of the whole mtDNA has been performed to date in endometriosis. In the present study, we attempted comprehensive scanning of somatic & germ-line mtDNA mutations in matched ectopic (endometriotic) and eutopic (normal) endometrial tissues from 32 endometriosis patients. For high stringent data quality control, we compared the entire mtDNA sequences of matched ectopic and eutopic endometrial tissues along with blood samples from the same patients. Mitochondrial haplogrouping was performed in 128 cases and 90 controls to identify its possible association with the risk of endometriosis. Moreover, also we analyzed tumor specific large-scale mtDNA deletions and mitochondrial microsatellite instability (MSI) in ectopic tissues to verify their role in the pathogenesis of endometriosis.

## Materials and Methods

### Sample Collection and Diagnosis

Matched ectopic and eutopic endometrial tissue samples were collected from thirty two pre-menopausal unrelated women of South Indian origin (Dravidian linguistic group) with moderate-severe (III–IV; rAFS [23]) endometriosis. The samples were collected from Infertility Institute and Research Centre (IIRC), Secundrabad, India (n = 7) and Vasavi Medical and Research Centre, Hyderabad, India (n = 25). Each pair of ectopic and eutopic endometrial tissues was collected from the same patient to minimize the genetic heterogeneity. Also, we collected blood samples from the same patients to confirm the heteroplasmic mutations in tumor tissues because it is possible that the apparent heteroplasmic mutation is attributable to the contamination of the surrounding non-tumor cells. All women had a trans-vaginal ultrasound scan (TVS) at screening followed by a laparoscopy to confirm the endometriosis stage (rAFS III = 10; IV = 22). All the patients had different manifestations of endometriosis such as peritoneal lesions, adhesions and endometrioma. Women with other ovarian cysts, adenomyosis, ovarian cancer, fibroids, and stage I and II endometriosis were excluded from the study. The aim was to focus on patients with more severe endometriosis (stage III and IV) because the more severe forms include ovarian cystic disease, which almost certainly has a different etiology to peritoneal forms, and the diagnosis is usually unequivocal, which is not the case for stage I and II. Tissue samples were frozen immediately in the operation room and stored at –80°C until isolation of genomic DNA was performed. For haplogrouping analysis peripheral blood samples were collected from 128 patients and 90 controls. The control group was selected from same clinical population as per the criteria of appropriate controls set [24]. Written informed consent was obtained from all participants. The Institutional Review Board of the Centre for Cellular and Molecular Biology (CCMB), Hyderabad, approved the study.

### Sequencing of Entire Mitochondrial Genome

DNA was isolated from all the samples by salting out method [25]. The entire mitochondrial genome mutations were analyzed by PCR-Sequencing analysis as per the protocols described by Bhanoori *et al.* [7]. Twenty-four pairs of overlapping primers (Table S1) were used to amplify the entire 16.569 kb mitochondrial genome. The generated DNA fragments vary in size from 801 bp to 1161 bp with an average of 895.96 bp. The amplified fragments totaled 21503 bp, 29.8% more than the mt-genome because of the overlapping regions.

### Mutational Analysis & Haplogrouping

The individual mtDNA sequences were compared against the Revised Cambridge Reference Sequence (rCRS) [26] using Auto Assembler-Ver 2.1 (Applied Biosystems, Foster City USA). Sequences were aligned by using CLUSTAL X and mutations were noted by using MEGA software ver 3.1. Independent sequencing readings were performed by three different individuals (SG, NRT & SP). Sequence variations found in both ectopic and matched eutopic mtDNA were scored as germ-line variations. Each was then checked against the Mitomap database. Those not recorded in the database were categorized as novel mtDNA variations, and those that appeared in the database were reported as polymorphisms or mutations. Any DNA sequence differences between ectopic and matched eutopic mtDNA were scored as somatic mtDNA mutations. All the somatic heteroplasmic mutations were crosschecked and confirmed by comparing the ectopic mtDNA sequences with the analogous eutopic as well as blood mtDNA sequences form the same patients. The level of heteroplasmy was calculated based on the ratio of the height of the mutant peak relative to the wild type plus mutant peak at the same nucleotide position in the sequencing results. Pathogenic potential of missense mutations was predicted by PolyPhen software (www.tux.embl-heidelberg.de/ramensky/polyphen.cgi). It works based on sequence comparison with homologous proteins. Profile scores (PSIC) were generated for the allelic variants which represents the logarithmic ratio of the likelihood of a given amino acid occurrence at a particular site relative to the likelihood of this amino acid occurrence at any site (background frequency). Generally, PSIC score differences >2 indicate a damaging effect, scores between 1.5 and 2 suggest that the variant is possibly damaging and scores <1.5 indicate that the variant is benign. Mitochondrial haplogroups were assigned to all samples based on updated comprehensive phylogenetic tree of global human mitochondrial DNA variation [27].

### Microsatellite Instability (MSI) Analysis

To study mtDNA MSI, 9 mononucleotide (nt 303, 311, 3566, 6692, 9478, 12385, 12418, 13231, and 16184), a dinucleotide (nt 514), and a trinucleotide (nt 12981) repeats at various nucleotide positions throughout mt-genome were analyzed by using sequencing data.

### mtDNA Deletion Analysis

The common 5 kb deletion (nt 8469 to nt 13447) in tumor tissue was analyzed by PCR method using forward primer mtFL8150 (5′-CCGGGGGTATACTACGGTCA-3′) and reverse primer mtRH14020 (5′-ATAGCTTTTCTAGTCAGGTT-3′). The PCR will produce either 894 bp fragment (4977 bp mtDNA deletion) and/or 536 bp fragment (5335 bp mtDNA deletion) or 5871 bp fragment (wild type mtDNA) based on the type of deletion. Both ectopic and matched eutopic tissues were included in this study.

### Statistical Analysis

Statistical analysis was performed using SPSS statistical package (ver 11.0). The frequency of each haplogroups among cases and controls were compared with Pearson χ^2^ or Fisher’s exact test. A *P*-value less than 0.05 was considered significant. Bonferroni correction was used to adjust the significance level of a statistical test to protect against Type I errors when multiple comparisons were being made. Since we have 39 mitochondrial haplogroups, the Bonferroni correction should be 0.05/39 = 0.00128. Therefore, a *P*-value less than 0.00128 was considered significant.

## Results

### Endometriosis Harbored Tumor Specific Somatic mtDNA Mutations

The first objective of this study was to evaluate whether endometriosis presented with the incidence of tumor specific somatic mtDNA mutations like many other tumors types. We identified 51 somatic mutations (Table 1) in which 45.1% (23/51) were located in the D-loop region, 17.6% (9/51) were in rRNA genes, 3.9% (2/51) were in tRNA genes and 33.3% (17/51) were present in protein coding region of mt-genome. The relative somatic mutation frequency in the D-loop region is 11.4-fold higher than the remaining region of mt-genome. Interestingly all the identified somatic mutations in protein coding region (n = 17) are novel. Overall, among the identified 51 somatic mutations 38 were point mutations, 2 were nucleotide deletions, 5 were nucleotide insertions and 6 were heteroplasmic mutations. Among six heteroplasmic mutations 5 (3 in 12S rRNA genes; 1 in 16S rRNA genes and 1 in ND4 gene) showed heteroplasmy in ectopic endometrium while in analogous eutopic endometrium they were homoplasmic wild type. Interestingly the remaining 1 heteroplasmic mutation, located in ND5 gene at nt 13603 showed heteroplasmy (A/G) in eutopic endometrium while homoplasmic mutant type (G/G) in analogous ectopic endometrium. To confirm the heteroplasmic mutations, we sequenced and compared the mtDNA of peripheral leukocytes along with the eutopic and ectopic tissues from the same patients, because heteroplasmy could be attributed to the contamination with the surrounding tumor or the morphologically normal tissue that have already undergone molecular changes. The blood mtDNA sequence pattern is fully consistent with eutopic tissue, indicating the purity of ectopic tissue.

**Table 1:** Somatic mtDNA mutations observed in Endometriosis patients^1^.

Gene/region		Nucleotideposition		Ref		Base change				Eut → Ect pattern	F	Codon &AA change	Reported/Novel^2^
						Bld		Eut	Ect				
D-loop		A56 del		A		A		A	–	HM → HM	1	NPCR	Novel
D-loop		A73G		A		A		A	G	HM → HM	2	NPCR	Reported
D-loop		C105 del		C		C		C	–	HM → HM	2	NPCR	Novel
D-loop		149 ins T		–		–		–	T	HM → HM	1	NPCR	Novel
D-loop		T195C		T		T		T	C	HM → HM	1	NPCR	Reported
D-loop		T204C		T		T		T	C	HM → HM	1	NPCR	Reported
D-loop		G207A		G		G		G	A	HM → HM	3	NPCR	Reported
D-loop		303–9 ins C		–		–		–	C	HM → HM	7	NPCR	Reported
D-loop		303–9 ins CC		–		–		–	CC	HM → HM	5	NPCR	Reported
D-loop		T310C		T		T		T	C	HM → HM	1	NPCR	Reported
D-loop		311-15 ins C		–		–		–	C	HM → HM	2	NPCR	Reported
D-loop		G316C		G		G		G	C	HM → HM	2	NPCR	Reported
D-loop		C394T		C		C		C	T	HM → HM	1	NPCR	Novel
D-loop		A438G		A		A		A	G	HM → HM	1	NPCR	Novel
D-loop		G709A		G		G		G	A	HM → HM	1	NPCR	Reported
12S rRNA		C775C/G		C		C		C	C/G	HM → HT	1	NPCR	Novel
12S rRNA		G811G/A		G		G		G	G/A	HM → HT	3	NPCR	Novel
12S rRNA		A1171A/G		A		A		A	A/G	HM → HT	10	NPCR	Novel
12S rRNA		A1383G		A		A		A	G	HM → HM	1	NPCR	Novel
16S rRNA		A1811G		A		A		A	G	HM → HM	1	NPCR	Reported
16S rRNA		C2005T		C		C		C	T	HM → HM	1	NPCR	Novel
16S rRNA		G2536G/A		G		G		G	G/A	HM → HT	30	NPCR	Novel
16S rRNA		A2706G		A		A		A	G	HM → HM	1	NPCR	Reported
16S rRNA		C2961T		C		C		C	T	HM → HM	1	NPCR	Novel
ND1		C3573T		C		C		C	T	HM → HM	3	L89L	Novel
ND2		C4799T		C		C		C	T	HM → HM	1	P110P	Novel
ND2		C5444A		C		C		C	A	HM → HM	1	F325L	Novel
tRNA^Cys^		G5821A		G		G		G	A	HM → HM	1	NPCL	Reported
COI		C7019T		C		C		C	T	HM → HM	1	Y372Y	Novel
CO1		T7372C		T		T		T	C	HM → HM	1	M490T	Novel
COII		A8116G		A		A		A	G	HM → HM	1	G177G	Novel
ATPase6		A8698G		A		A		A	G	HM → HM	1	M58V	Novel
ATPase6		C9061T		C		C		C	T	HM → HM	1	L179L	Novel
COIII		C9650T		C		C		C	T	HM → HM	1	H148H	Novel
ND4		T11399C		T		T		T	C	HM → HM	1	L204L	Novel
ND4		C11542T		C		C		C	T	HM → HM	1	F261F	Novel
ND4		T11544T/A		T		T		T	T/A	HM → HT	7	L262H	Novel
ND4		A11707G		A		A		A	G	HM → HM	1	M316M	Novel
ND4		T11792G		T		T		T	G	HM → HM	1	S345A	Novel
ND5	C12398T		C		C		C		T	HM → HM	1	T21I	Novel
ND5	C12867T		C		C		C		T	HM → HM	1	I177I	Novel
ND5	A/G13603G		A		A/G		A/G		G	HT → HM	32	S423G	Novel
tRNA^Thr^	C15926T		C		C		C		T	HM → HM	2	NPCR	Novel
D-loop	G16145A		G		G		G		A	HM → HM	3	NPCR	Reported
D-loop	C16172T		C		C		C		T	HM → HM	2	NPCR	Reported
D-loop	A16182C		A		A		A		C	HM → HM	1	NPCR	Reported
D-loop	T16189C		T		T		T		C	HM → HM	5	NPCR	Reported
D-loop	C16325T		C		C		C		T	HM → HM	1	NPCR	Reported
D-loop	16351ins C		–		–		–		C	HM → HM	3	NPCR	Novel
D-loop	T16362C		T		T		T		C	HM → HM	2	NPCR	Reported
D-loop	T16519C		T		T		T		C	HM → HM	1	NPCR	Reported

1 Total number of mutations: 51; ^2^Novel mutations: 31, Reported mutations: 20;

Ref, Cambridge reference sequence; Bld, Blood; Eut, Eutopic endometrium;

Ect, Ectopic endometrium; F, Frequency of mutations; HM, Homoplasmic mutation;

HT, Heteroplasmic mutation; NPCR, Non Protein Coding Region;

### Effect of Somatic Mutations on Mitochondrial Function

Among the 17 somatic mutations identified in mtDNA protein coding region, 7 (41.2%) were missense that may have the potential to cause defects in the oxidative phosphorylation system (OXPHOS).

**Figure 1:**
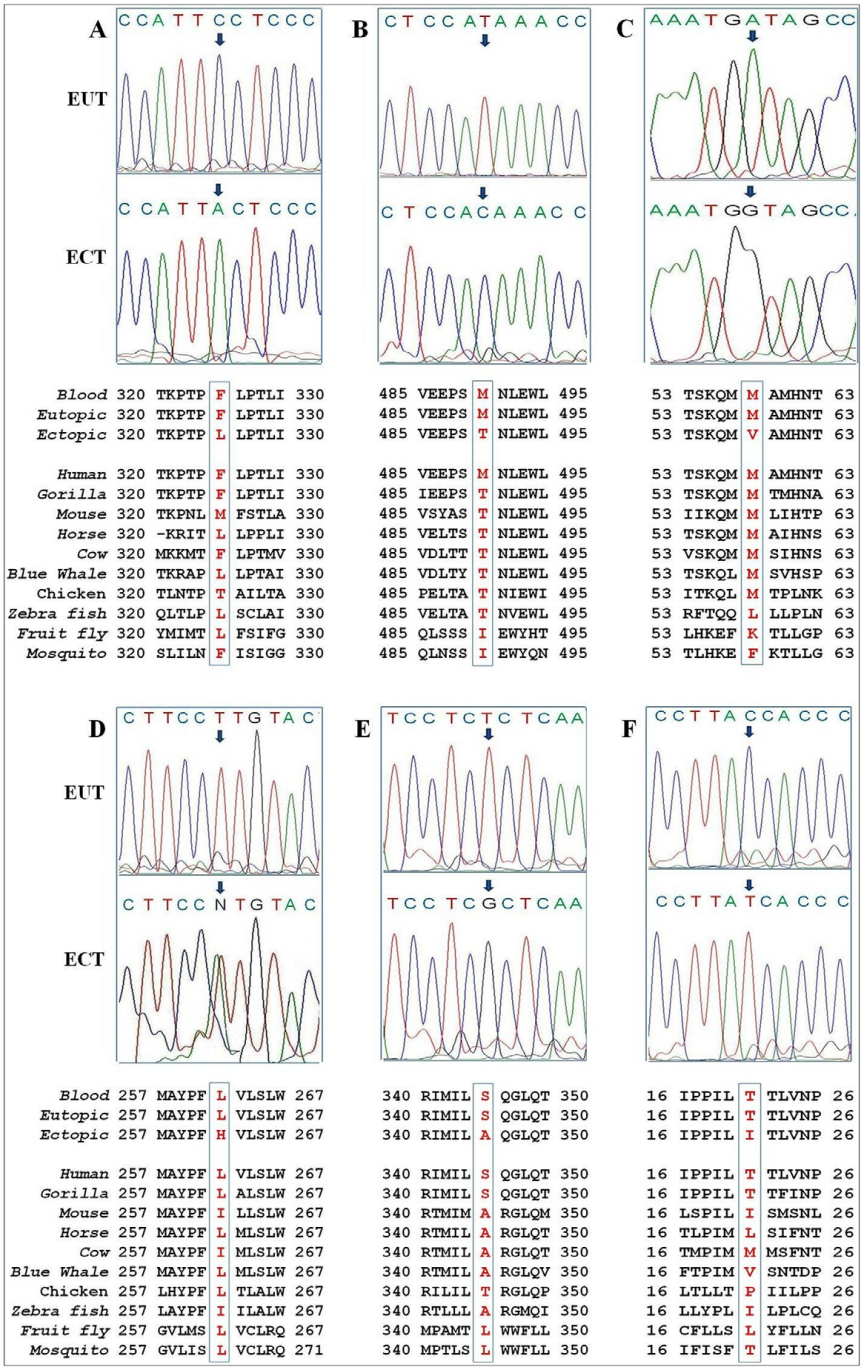
Novel somatic missense mtDNA mutations observed in endometriosis patients. (A) The C5444A transversion leads to F325L substitution in the ND2 subunit; (B) The T7372C transition leads to M490T substitution in the COI subunit; (C) The A8698G transition leads to M58V substitution in the ATPase6 subunit; (D) The T11544T/A transversion heteroplasmy leads to L262H substitution in the ND4 subunit; (E) The T11792G transversion leads to S345A substitution in the ND4 subunit; (F) The C12398T transition leads to T21I substitution in the ND5 subunit.

The C5444A in ND2 gene (Figure 1A) and the T7372C in COI gene (Figure 1B) result in substitution from phenylanine to leucine and methionine to threonine respectively. These two point mutations are missense mutations, but located in poorly conserved region of proteins. Thus presumably they have no impact on protein structure and/or function. The Profile score (PSIC) difference was 0.031 for C5444A and 0.184 for T7372C, thus they were predicted to be benign by PolyPhen analysis. The A8698G mutation results in substitution from methionine to valine at codon 58 of the ATPase 6 (Figure 1C). This may be harmful missense mutation because this position is highly conserved among higher vertebrates (PSIC = 1.893). The T11544T/A heteroplasmic mutation (48–51% heteroplasmy) was identified in ectopic endometrium of 7 patients. It causes a substitution from leucine to histidine at codon 262 of the ND4 gene (Figure 1D). The location of this mutation is evolutionally highly conserved (L or I) among most of the eukaryotes, thus it may be a potentially harmful missense mutation (PSIC = 2.050).

The T11792G in ND4 gene and the C12398T in ND5 gene result in substitution from serine to alanine and threonine to isoleucine respectively (Figure 1E–F). These two point mutations may not be harmful missense mutations as they occur at poorly conserved region of proteins. PolyPhen analysis predicted both of these variants as benign (PSIC = 0.458 for T11792G & not applicable for C12398T).

### Association of ‘A13603G’ Mutation of ND5 with Endometriosis

**Figure 2:**
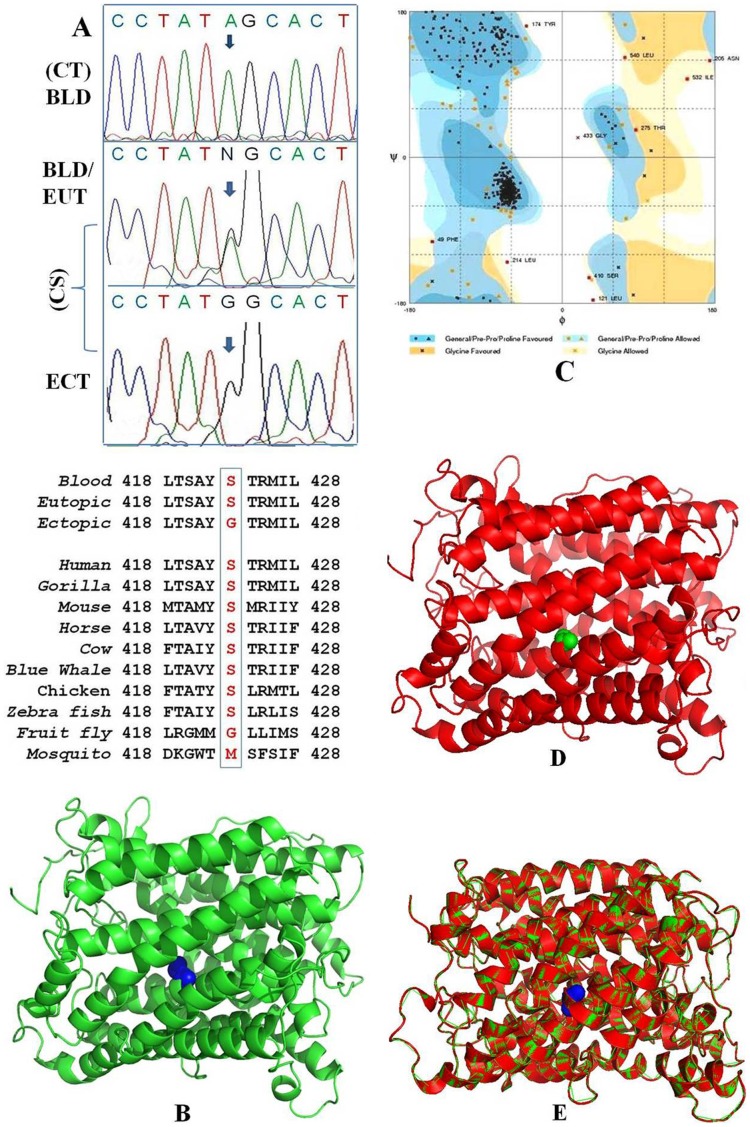
The ‘A13603G’ mutation of ND5 gene. (A) Sequence analysis of ‘A13603G’ mutation using a forward primer. Homoplasmic wild (13603A) and mutant alleles (13603G) appear as single peaks whereas heteroplasmic allele (13603A/G) as double peak. Evolutionary conservation analysis of mutation is also shown. CT: Control, CS: Case, BLD: Blood, EUT: Eutopic endometrium, ECT: Ectopic endometrium; (B) Native structure of ND5 subunit: ‘serine 423’ is shown as a blue sphere; (C) Ramachandran plot showing the structural accuracy of native structure of ND5 subunit: all the amino acids are in the allowed region except proline and glycine; (D) Mutated structure of ND5 subunit: ‘glycine 423’ is shown as a green sphere; (E) Superimposition of native (green) and mutant (red) structures of ND5 subunit.

We identified a novel somatic missense ‘A→G transition’ mutation at the nt 13603 (Figure 2A) in ectopic endometrium of all the patients (n = 32) which causes a substitution from serine to glycine at the codon 423 of ND5 gene. It may be a potentially harmful mutation as it occurs in highly conserved functional domain of the peptide (PSIC = 2.193). Interestingly, eutopic endometrium & peripheral leukocytes of all the patients showed different levels heteroplasmy (A/G; 40–80%) at this locus, while their ectopic endometrium showed homoplasmic mutant allele (G/G). The level of heteroplasmy (mutation load) was inconsistent between different tissues of same patients. Overall, eutopic endometrium showed high mutationload than the analogous peripheral leukocytes. Furthermore, we did not observe this mutation among age matched controls from the same ethnic group. These results emphasize the correlation between ‘A13603G’ mtDNA mutation and risk of endometriosis.

### Structure Prediction and *In Silico* Functional Analysis of ‘A13603G’ of ND5

We compared all 164 sequence depositions of human ND5 gene available from the NCBI database using Clustal X software for similarities. Using protein prediction servers we found 1EHK as template structural sequence for the ND5 target sequence. There is no full pledge crystallized structure available for the ND5. Template sequence has 30% identity to the ND5. We performed the homology modeling and generated 40 structures for ND5 protein using modeller9V9 software [28]. Of these we considered one structure (Figure 2B) with low energy value (geometrically favorable). The structural accuracy of the selected structure was evaluated by Procheck software [29] (Figure 2C). It indicates that the generated structure was 98% geometrically favorable and can be reliable for further studies. Then we have mutated the native structure at amino acid position 423 by glycine (Figure 2D) and superimposed the native and mutated structures (Figure 2E) to find out the structural variations. Our results showed no structural difference between native and mutated forms. Serine is a polar amino acid, while glycine is hydrophobic, both of them, however belongs to the class of the smallest amino acids, with short side chain. It seems that the presence of small amino acids in this position is crucial for the maintenance of the protein structure without causing destabilization. The exact role of this mutation in pathogenesis of endometriosis is currently unknown. Further extensive biochemical and molecular functional studies are necessary to confirm the role of this mutation in endometriosis.

In addition, we identified two somatic mutations in tRNA coding genes. The G5821A mutation (Figure S1A ) in the tRNA^Cys^ gene interrupts conserved Watson- Crick base pairing in the aminoacyl acceptor stem and is likely to alter the secondary or tertiary structure of the cloverleaf. The C15926T mutation (Figure S1B) in the tRNA^Thr^ gene newly establishes Watson- Crick base pairing in the anticodon loop. Thus it may alter the overall structural stability of tRNA^Thr^.

### Germ-line mtDNA Mutations are Common in Endometriosis

We observed 53 novel and 530 reported germ-line mtDNA variations among the patients. Among 53 novel mutations, 11 are missense mutations (Table 2), 22 are synonymous mutations (Table S2) and remaining (n = 20) are present in non protein coding region of mt-genome (Table S3). The overall frequency of novel germ-line mutations was 16.98% (9/53) in the D-loop and 83.02% (44/53) in the coding regions. Forty one of the 53 (77.35%) novel variations occurred only once, whereas the remaining variations showed differential frequency. All the above 53 novel variations are completely absent in controls.

**Table 2:** Novel missense mtDNA mutations observed in endometriosis patients^1^.

Gene/region	Nucleotide position	Ref	Base change			Germline/Somatic^2^	F	Codon &AA change	Con^3^
			Bld	Eut	Ect				
ND2	T4509C	T	C	C	C	germ-line	1	F14L	PC
ND2	A4701G	A	G	G	G	germ-line	2	N78D	HC
ND2	C5444A	C	C	C	A	somatic	1	F325L	PC
ND2	C5445T	C	T	T	T	germ-line	1	L326F	CN
COI	T7372C	T	T	T	C	somatic	1	M490T	PC
COII	G7775A	G	A	A	A	germ-line	2	V64I	CN
COII	T7836C	T	C	C	C	germ-line	1	L84P	HC
ATPase6	A8698G	A	A	A	G	somatic	1	M58V	CN
ATPase6	A8704G	A	G	G	G	germ-line	1	M60V	CN
ND4	T11544T/A	T	T	T	A	somatic	7	L262H	HC
ND4	T11792G	T	T	T	G	somatic	1	S345A	PC
ND5	C12398T	C	C	C	T	somatic	1	T21I	PC
ND5	T12448A	T	A	A	A	germ-line	1	S38T	PC
ND5	C12498T	C	T	T	T	germ-line	4	S38T	CN
ND5	T13154C	T	C	C	C	germ-line	1	I273T	PC
ND5	T13543C	T	C	C	C	germ-line	1	Y403H	CN
ND5	A/G13603G	A	A/G	A/G	G	somatic	32	S423G	HC
ND5	T13820C	T	C	C	C	germ-line	1	F495S	PC

1 Total number of mutations: 18; ^2^Germ-line mutations: 11, Somatic mutations: 7; ^3^Conservation; Ref, Cambridge reference sequence.

Bld, Blood; Eut, Eutopic endometrium; Ect, Ectopic endometrium; F, Frequency of mutations; PC, Poorly conserved; HC, Highly conserved; CN, Conserved.

Among 530 reported germ-line mutations, 73 (13.8%) are missense mutations, 236 (44.5%) are synonymous mutations and remaining are located in non protein coding region of mt-genome (data not shown). In overall, 151 (28.5%) are located in the D-loop region. The relative germ-line mutation frequency in the D-loop region is 6.5-fold higher than the remaining region of mt-genome.

### Association of Haplogroup M5 with Endometriosis Risk

We identified 39 mtDNA haplogroups among the patients and controls (Table 3). We found a significant association between haplogroup M5 and endometriosis risk (*P* = 0.00069). Further we tested the correlation between identified macro-haplogroups and endometriosis incidence. We found no statistical significant difference between cases and controls.

**Table 3:** Mitochondrial haplogroup distribution in endometriosis patients and controls^1^.

Haplogroup	Cases	Controls	*P*-value^2^	χ^2^-value	Odds ratio	95% CI
H2a2a	4 (3.1)	2 (2.2)	0.69568	0.153	0.7045	0.1262 to 3.9314
J1	1 (0.8)	4 (4.4)	0.11443	2.492	5.907	0.6491 to 53.7591
M	1 (0.8)	3 (3.3)	0.21702	1.524	4.3793	0.4481 to 42.7975
M18	1 (0.8)	5 (5.6)	0.05778	3.6	7.4706	0.8576 to 65.0753
M2	9 (7.0)	4 (4.4)	0.44126	0.593	0.615	0.1834 to 2.0625
M3	7 (5.4)	3 (3.3)	0.47644	0.507	0.5961	0.1499 to 2.3702
M30	10 (7.8)	9 (10)	0.60199	0.272	1.3111	0.5101 to 3.3696
M31	0 (0)	1 (1.1)	0.29427	1.1	–	–
M33	1 (0.8)	0 (0)	0.37109	0.8	–	–
M34	0 (0)	2 (2.2)	0.13801	2.2	–	–
M35	5 (3.9)	2 (2.2)	0.49115	0.474	0.5591	0.106 to 2.9479
M36	1 (0.8)	0 (0)	0.37109	0.8	–	–
M37	0 (0)	2 (2.2)	0.13801	2.2	–	–
M39	5 (3.9)	1 (1.1)	0.2105	1.568	0.2764	0.0317 to 2.4071
M4	7 (5.4)	5 (5.6)	0.94957	0.004	1.0168	0.3122 to 3.3114
M40	0 (0)	1 (1.1)	0.29427	1.1	–	–
M41	0 (0)	1 (1.1)	0.29427	1.1	–	–
M42	1 (0.8)	0 (0)	0.37109	0.8	–	–
M49	3 (2.3)	0 (0)	0.12937	2.3	–	–
**M5**	25 (19.5)	3 (3.3)	**0.00069**	11.511	0.1421	0.0415 to 0.4867
M52	1 (0.8)	0 (0)	0.37109	0.8	–	–
M58	0 (0)	1 (1.1)	0.29427	1.1	–	–
M6	6 (4.7)	7 (7.8)	0.38053	0.769	1.7149	0.5564 to 5.2851
M64	0 (0)	1 (1.1)	0.29427	1.1	–	–
R	8 (6.2)	2 (2.2)	0.16752	1.905	0.3409	0.0707 to 1.6447
R2	0 (0)	1 (1.1)	0.29427	1.1	–	–
R7	0 (0)	3 (3.3)	0.06928	3.3	–	–
R30	2 (1.6)	0 (0)	0.2059	1.6	–	–
R31	1 (0.8)	0 (0)	0.37109	0.8	–	–
R5	1 (0.8)	4 (4.4)	0.11443	2.492	5.907	0.6491 to 53.7591
R6	4 (3.1)	6 (6.7)	0.25023	1.322	2.2143	0.6064 to 8.0855
R8	1 (0.8)	1 (1.1)	0.82837	0.047	1.427	0.0881 to 23.1187
T1	1 (0.8)	0 (0)	0.37109	0.8	–	–
T2	1 (0.8)	0 (0)	0.37109	0.8	–	–
U1	0 (0)	1 (1.1)	0.29427	1.1	–	–
U2	11 (8.6)	12 (13.3)	0.31514	1.009	1.6364	0.6877 to 3.8937
U5	1 (0.8)	0 (0)	0.37109	0.8	–	–
U7	8 (6.2)	3 (3.3)	0.34684	0.885	0.5172	0.1334 to 2.0057
W	1 (0.8)	0 (0)	0.37109	0.8	–	–

1 Haplogroups observed = 39; ^2^Banferoni correction for *P*-value = 0.05/39 = 0.00128.

### Microsatellite Instability (MSI) is Less Frequent in Endometriosis

Somatic homoplasmic insertions were found in nt 303–309 and 311–315 poly C tracts only. In addition we found a T to C substitution at nt 16189 which resulted in a stretch of 10 ‘C’s in the region. None of the remaining MS loci showed obvious insertion or deletion of ‘C’s.

### Tumor Specific Deletions are Absent in Endometriosis

The common 5 kb tumor-specific deletion (nt 8469 to nt 13447) was analyzed in ectopic endometrial tissues by PCR method. This method is sensitive enough to detect 0.01% of deleted mtDNA. Deletions were not detected in any of the ectopic tissues.

## Discussion

A new role of ROS as second messenger of cellular proliferation has been reported. According to this, normal cell proliferation correlated with production of endogenous ROS through the activation of growth-related signaling pathways [30]. Modulation of ROS production may control tumor cell proliferation [31]. The mitochondrial ETC is a major source for ROS generation. Mutations in mitochondrial DNA have been found to increase endogenous ROS production and enhance metastatic potential of tumor cells [16]. It has been suggested that ROS or free radicals may increase growth and adhesion of endometrial cells in the peritoneal cavity, promoting endometriosis and infertility [32]–[33]. However, the published studies on the association between oxidative stress and endometriosis have been inconsistent. Some of the studies have found a positive association between oxidative stress and endometriosis [14], [34] whereas others have not found an association [35]–[36]. In addition, increased oxidative stress and mtDNA deletions have been observed in endometriotic tissues from patients [37]. All these observations strongly correlate the mitochondrial malfunction with the pathogenesis of endometriosis.

Previously investigators have screened the whole mt-genome in a variety of human diseases including cancer [22]. However none of the investigators have reported whole mtDNA mutations in endometriosis. The current sequencing based analysis of the entire mt-genome demonstrates that endometriotic tissues harbor a plethora of tumor-specific somatic mtDNA mutations much like other tumors. Our study provides, for the first time, an insight into the prevalence and distribution of entire mtDNA mutations in endometriosis. Association between 3 reported germ-line mtDNA mutations (A10398G, G13708A and T16189C) and endometriosis have recently been reported [38]. Although interpretation of linking germ-line mtDNA mutations to tumor can be confounded by the high background frequency of functional mtDNA polymorphisms, studies of somatic mtDNA mutations can be more definitive since the tumor cell should have the neoplastic mtDNA mutation while the normal tissue should not. Moreover, if germ-line mtDNA mutations induce oncogenic transformation, all the offspring of a mother carrying such mutations should develop tumors due to the maternal inheritance of mtDNA, but no bias toward maternal inheritance of tumor development has been reported. In the present study we reported a comprehensive study of somatic and germ-line mtDNA mutations in endometriosis.

Previously several reporters have shown the prevalence of somatic mtDNA mutations in various types of tumors [22]. The high frequency of somatic mutations was attributed to artifacts instead of real somatic events [39]. A critical reassessment of the reported somatic mutations in tumors was carried out using a phylogenetic method and found that many of these investigations were affected by flawed data which were mainly caused by sample crossover and contamination [39]. In the present study we maintained high stringent data quality control to avoid these problems. Of the 51 somatic mutations that we found, 20 (17 in the D-loop and 3 in non-D loop areas) have been previously reported in other cancer types (Table 1) suggesting that these sites are susceptible to mutation in a variety of human malignancies. Our observations underpin the previous findings that somatic mtDNA mutations in tumors are concentrated in the D-loop and that the D-310 region is a mutation hot spot. The sequence between nt 303–315 is highly conserved and the length variation or mutations in this region affects regulation of mtDNA replication. The 5 mutations that we and others observed in this conserved region in tumor specimens may therefore reflect neoplastic initiation.

The role of somatic mtDNA mutations in tumor progression has not been investigated. Mutations in the conserved regions, origins of replication, promoters or transcription factor binding sites, may affect the total amount of mitochondrial transcripts and mature proteins. Eventually, the overall OXPHOS activity of the mitochondria may be affected. We observed 7 somatic missense mutations that led to changes in amino acids (Table 1). All these mutations are novel and most of them are located in highly conserved regions of different mitochondrial genes. Each of these mutations may have functional significance, but more extensive biochemical and molecular studies are necessary to determine their effects on energy metabolism in the tumor cells. Interestingly, the A13603G (S423G) of ND5 gene was observed in ectopic endometrium of all patients (n = 32) while completely absent in matched eutopic endometrium and peripheral leukocytes of the same patients. More specifically, mtDNA of blood and eutopic endometrium of all patients showed different levels of heteroplasmy (A/G; 40 - 80%) at this locus while the analogous ectopic endometrium carried exclusively mutant mtDNA (homoplasmy) without any co-existing normal mtDNA (heteroplasmy), as is found in mitochondrial genomic degenerative diseases [18], [22]. This may be consequence of ‘replicative segregation’ – a process in which with repeated cell divisions (clonal expansion) the percentage of mutant and normal mtDNAs can drift until it reaches either pure mutant or normal homoplasmy (Figure S2). These findings support the idea that pathological mtDNA mutations are particularly deleterious in specific cell types, which can explain some of the tissue-specific aspects of mitochondrial DNA diseases. Interestingly, all control group individuals carried homoplasmic wild allele (A/A) at this locus. These observations emphasize the role of A13603G in pathogenesis of endometriosis. We predicted mitochondrial ND5 protein structure for the first time and observed no structural difference between native and mutated forms (Figure 2). Further studies are required to elucidate the exact pathogenic role of this mutation in endometriosis.

Several mitochondrial mutations have been reported in association with various human diseases. However, very few of them have been confirmed as markers. For LHON disease, three markers have been reported in approximately 95% of all patients while their frequency is 0% in controls (www.mitomap.org). Interestingly, in our study we identified 100% incidence of A13603G novel missense somatic mutation in ectopic endometrium and 0% incidence in eutopic edometrium and peripheral leukocytes of patients. However, further studies are required considering different ethnic groups to elucidate the A13603G mutation as marker of endometriosis.

Most of the ectopic tissues had more than one somatic mutation. Specifically, of the 32 ectopic tissues with somatic mutations, 30 (93.75%) had two or more, 16 had three or more (50%) and 5 had six or more (15.6%) mutations (Table 1). Multiple mtDNA mutations are common in most of the human tumors [40]. Transcription of mitochondrial genome produces two polycistronic primary transcripts that are processed by endonuclease to yield the mature rRNA, tRNA, and mRNA molecules. Thus, mutations anywhere in the genome affecting the folding and secondary structure of the RNA precursors are potentially detrimental to RNA processing. However, most of the somatic mtDNA mutations in tumor may represent passenger mutations that do not play any primary role in tumorigenesis.

We identified a huge number (novel: 53; reported: 530) of germ-line mtDNA mutations in endometriosis patients. Of 530 reported germ-line mutations, 151 (28.4%) are present in the D-loop, an area that acts as a promoter for both the heavy and light strands of mtDNA which contains essential transcription and replication elements. The frequency of novel germ-line variations in coding regions [83.01% (44/53)] is much higher than in the D-loop [16.98% (9/53)]. Although we cannot be sure, this may simply reflect the fact that the D-loop has been more intensively investigated than the coding regions.

Mutations in different mitochondrial gene complexes have been shown to contribute to tumorigenicity through ROS generation in several tumor types [41]. The high incidence of missense mutations in complex I has been reported in several tumors [41]–[42]. Our results are consistent with this finding. We observed 5/7 somatic and 8/11 novel germ-line missense mutations in complex I genes (Table 1 & 2). Mitochondrial OXPHOS produces most of the cellular ROS at complexes I and III, by the transfer of an unpaired electron to O_2_, it produces superoxide radicals (O_2_^−^). Inhibition of electron flux through the OXPHOS causes retention of electrons on complex I & III electron carriers which make direct availability of electrons to O_2_ to generate O_2_^−^. Mitochondrial ROS can then damage mitochondrial enzymes, lipids and mutagenize the mtDNA.

Indian subcontinent harbors the largest diversity of mtDNA haplogroups after Africa [43]. The mtDNA haplogroups mainly fall into three macro-groups, designated as L, M and N, which exhibits distinct geographic distribution. L is the oldest macro-haplogroup, restricted to Africans and consists of haplogroups L0, L1, L2, L3, L4, L5, L6 and L7, of which L3 radiated out of Africa in the form of macro-haplogroups M and N around ∼60,000 ybp [43]. Among the two macro-haplogroups M and N, M is more prevalent in Indian subcontinent. The haplogroups are associated with region-specific mtDNA sequence variations as a result of genetic drift and/or adaptive selection for an environment-favored mitochondrial function [44]. Several investigators have showed association between different mtDNA haplogroups and various diseases including cancer [20]–[21]. Difference in redox signaling as a consequence of haplogroup-associated OXPHOS capacity has been suggested as the molecular mechanism involved in the haplogroups-associated phenotypes [45]. In the present study we report for the first time an association between haplogroup M5 and endometriosis in South Indian population.

Although not a primary aim of our investigation, also we analyzed MSI of mtDNA in ectopic endometrium of patients. Contradictory observations have been reported regarding mtDNA MSI in different tumors [46]–[47]. The methods used to detect the MSI were either fragment size analysis or RFLP analysis, which may not be the most accurate means because of the interference of shadow bands and the possibility of incomplete digestion, respectively. Differences in the frequency of MSI may be related to differences in methodology used, as well as the loci evaluated. We investigated the mtDNA MSI by direct sequencing method, and found insertions only in the D loop regions of nt 303–309 and nt 311–315. MSI was not detected in any of the other 10 selected STR regions except at nt T16189C. These observations indicate that the regions of nt 303–309 and nt 311–315 are probably mutation hot spots rather than the reflection of true MSI.

Tumor-specific large scale mtDNA deletions (4977 bp) have been detected in various diseases including cancer [48]–[49]. mtDNA deletions occur at a region that is flanked by short direct repeat sequences (class I deletions) or by imperfect repeats containing a few mismatches (class II deletions) which is usually lost during the deletion process [50]. Defects in mtDNA replication caused by inappropriate alignment of direct repeats [51] and/or altered repair of mtDNA damage [52] may be responsible for mtDNA deletions. Tumor-specidic mtDNA deletions have also been reported in endometriotic tissues [37]. However in the present study we did not found any large-scale mtDNA deletions both in ectopic and eutopic endometrium of the patients.

In conclusion, our study confirms the strong association between mtDNA variations and endometriosis risk. In addition, we found that haplogroup M5 exhibited an increased risk of endometriosis incidence. To our knowledge, this is the first report of complete mt-genome sequencing and haplogrouping in endometriosis. Present study gives a cumulative evidence that mtDNA is a rational biomarker for the detection of endometriosis. Further investigation is warranted as to the functional implications of identified mutations in endometriosis.

## Supporting Information

Figure S1 **Somatic mtDNA mutations observed in tRNA genes of endometriosis patients.** (A) The G5821A mutation in tRNA^Cys^; (B) The C15926T mutation in tRNA^Thr^.
(TIF)

Figure S2 **Differential mutation load shown by ‘A13603G’ mutation of ND5 gene.** (A) Homoplasmic wild allele: 13603A; (B) 48% heteroplasmy: 13603A/G; (C) 54% heteroplasmy: 13603A/G; (D) 57.1% heteroplasmy: 13603A/G; (E) 62.5% heteroplasmy: 13603A/G; (F) Homoplasmic mutant allele: 13603G.
(TIF)

Table S1 **Primers used in this study for whole mitochondrial genome sequencing.**
(DOC)

Table S2 **mtDNA novel synonymous mutations observed in endometriosis patients.**
(DOC)

Table S3 **mtDNA novel non-protein coding region mutations observed in endometriosis patients.**
(DOC)
