# Supplementary figures and images for: Mitochondrial Genome Variations in Advanced Stage Endometriosis: A Study in South Indian Population
Source: PLoS One. 2012 Jul 17;7(7):e40668. doi: 10.1371/journal.pone.0040668 (PMC3398934; doi:10.1371/journal.pone.0040668)

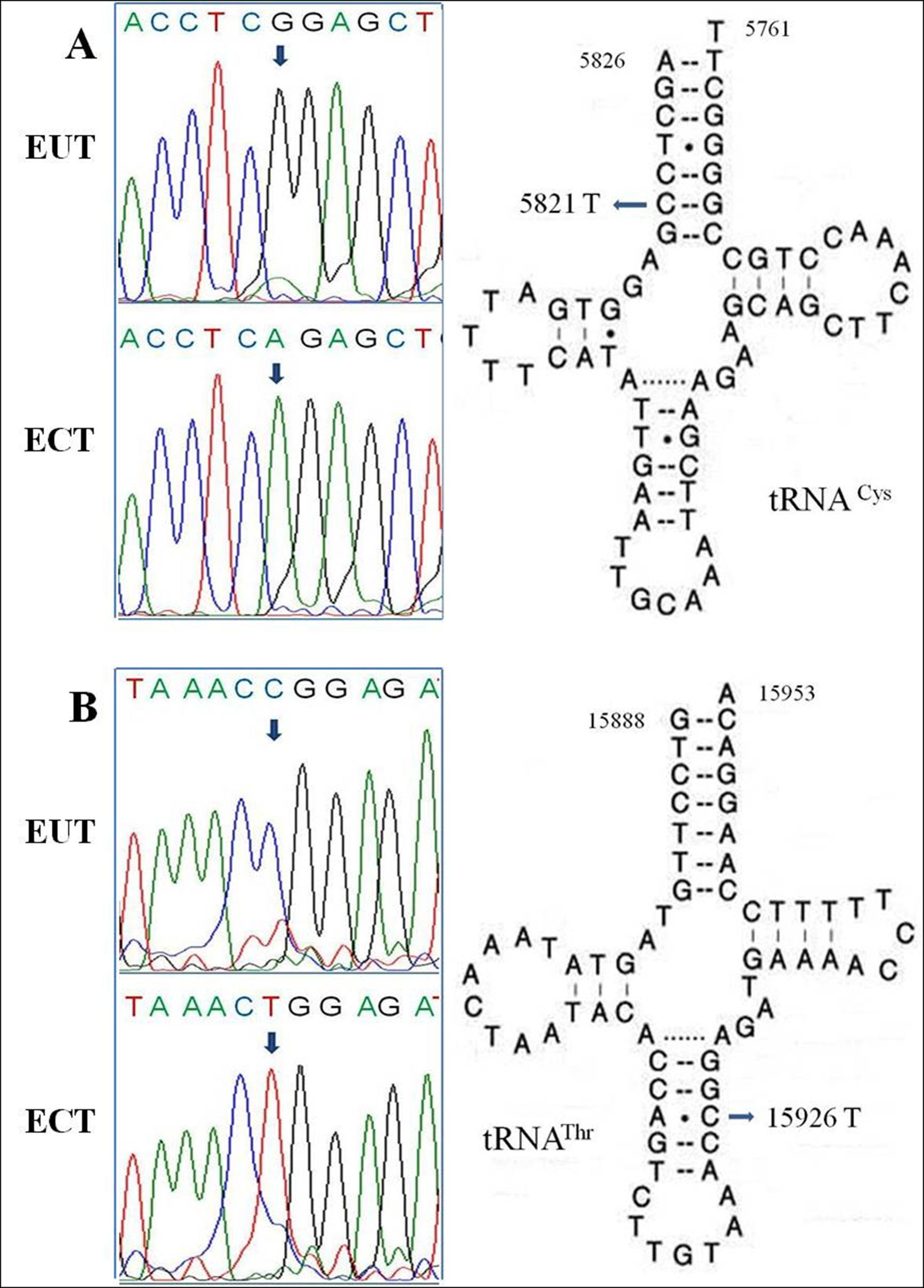

Supplement: Figure S1 — Somatic mtDNA mutations observed in tRNA genes of endometriosis patients. (A) The G5821A mutation in tRNACys; (B) The C15926T mutation in tRNAThr. (TIF) [file pone.0040668.s001.tif]

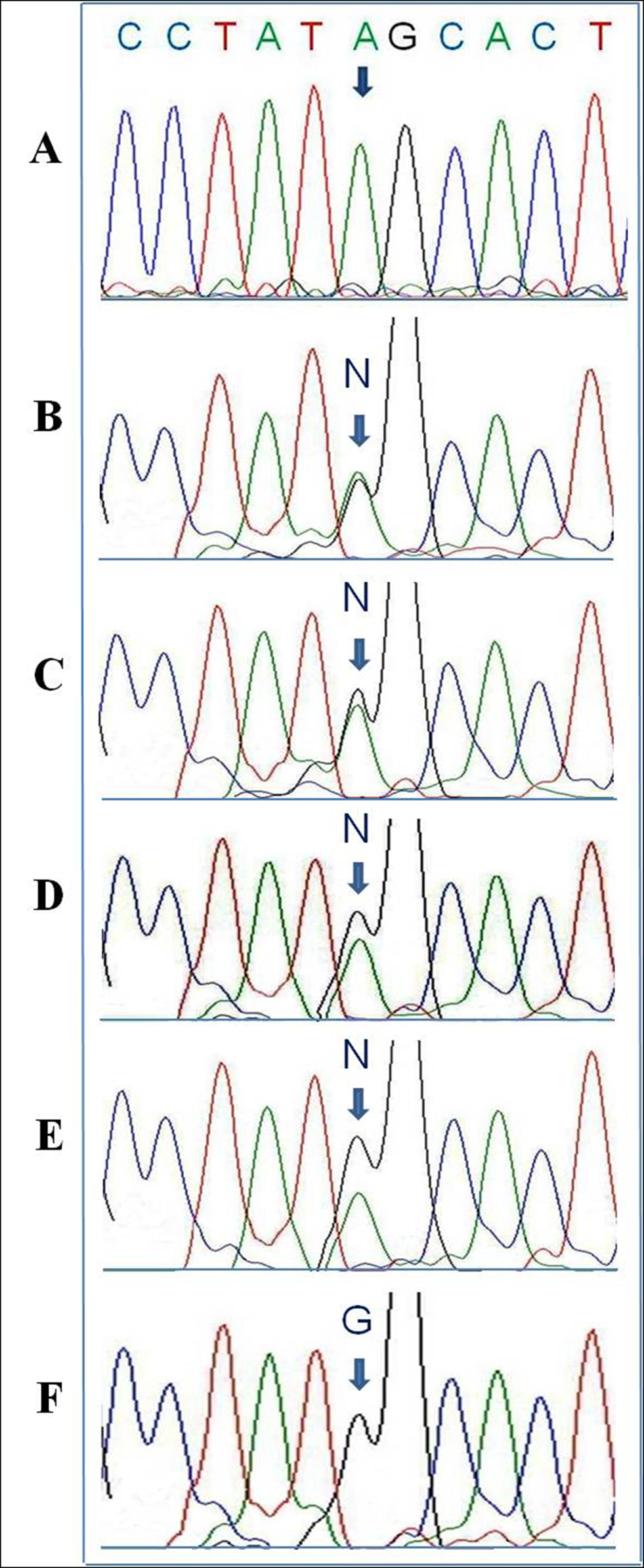

Supplement: Figure S2 — Differential mutation load shown by ‘A13603G’ mutation of ND5 gene. (A) Homoplasmic wild allele: 13603A; (B) 48% heteroplasmy: 13603A/G; (C) 54% heteroplasmy: 13603A/G; (D) 57.1% heteroplasmy: 13603A/G; (E) 62.5% heteroplasmy: 13603A/G; (F) Homoplasmic mutant allele: 13603G. (TIF) [file pone.0040668.s002.tif]
