# Supplementary material for: Mitochondrial Genome Variations in Advanced Stage Endometriosis: A Study in South Indian Population
Source: PLoS One. 2012 Jul 17;7(7):e40668. doi: 10.1371/journal.pone.0040668 (PMC3398934; doi:10.1371/journal.pone.0040668)
Supplement: Table S1 — Primers used in this study for whole mitochondrial genome sequencing. (DOC) [file pone.0040668.s003.doc]

**Table S1**

**Primers used in this study for whole mitochondrial genome** sequencing

| **S. No** | **Primer**  **set** | **Primer sequence 5'**→**3'** | **Nucleotide position** | **Overlap**  **(bp)** | **Amplicon size (bp)** | **Tan^1^ (^o^C)** |
| --- | --- | --- | --- | --- | --- | --- |
| 1 | 1F  1R | CTCCTCAAAGCAATACACTG  TGCTAAATCCACCTTCGACC | 592 -  1430 | 202 | 839 | 58 |
| 2 | 2F  2R | CGATCAACCTCACCACCTCT  TGGACAACCAGCTATCACCA | 1226 -  2026 | 204 | 801 | 58 |
| 3 | 3F  3R | GGACTAACCCCTATACCTTCTGC  GGCAGGTCAATTTCACTGGT | 1830 -  2688 | 196 | 859 | 55 |
| 4 | 4F  4R | AAATCTTACCCCGCCTGTTT  AGGAATGCCATTGCGATTAG | 2480 -  3365 | 208 | 886 | 56 |
| 5 | 5F  5R | TACTTCACAAAGCGCCTTCC  ATGAAGAATAGGGCGAAGGG | 3150 -  3980 | 215 | 831 | 60 |
| 6 | 6F  6R | TGGCTCCTTTAACCTCTCCA  AAGGATTATGGATGCGGTTG | 3777 -  4679 | 203 | 903 | 56 |
| 7 | 7F  7R | ACTAATTAATCCCCTGGCCC  AATGGGGTGGGTTTTGTATG | 4466 -  5443 | 213 | 978 | 58 |
| 8 | 8F  8R | CTAACCGGCTTTTTGCCC  ACCTAGAAGGTTGCCTGGCT | 5238 -  6050 | 205 | 813 | 61 |
| 9 | 9F  9R | GAGGCCTAACCCCTGTCTTT  ATTCCGAAGCCTGGTAGGAT | 5835 -  6661 | 215 | 827 | 61 |
| 10 | 10F  10R | CTCTTCGTCTGATCCGTCCT  AGCGAAGGCTTCTCAAATCA | 6450 -  7334 | 211 | 885 | 55 |
| 11 | 11F  11R | ACGCCAAAATCCATTTCACT  CGGGAATTGCATATGTTTTT | 7129 -  8114 | 205 | 986 | 55 |
| 12 | 12F  12R | ACGAGTACACCGACTACGGC  TGGGTGGTTGGTGTAAATGA | 7908 -  8816 | 206 | 909 | 58 |
| 13 | 13F  13R | TTTCCCCCTCTATTGATCCC  GTGGCCTTGGTATGTGCTTT | 8602 -  9416 | 214 | 815 | 55 |
| 14 | 14F  14R | CCCACCAATCACATGCCTAT  TGTAGCCGTTGAGTTGTGGT | 9211 -  10149 | 205 | 939 | 55 |
| 15 | 15F  15R | TCTCCATCTATTGATGAGGGTCT  AATTAGGCTGTGGGTGGTTG | 9967 -  10858 | 182 | 892 | 55 |
| 16 | 16F  16R | GCCATACTAGTCTTTGCCGC  TTGAGAATGAGTGTGAGGCA | 10653 -  11511 | 205 | 859 | 58 |
| 17 | 17F  17R | TCACTCTCACTGCCCAAGAA  GGAGAATGGGGGATAGGTGT | 11295 -  12095 | 216 | 801 | 55 |
| 18 | 18F  18R | TATCACTCTCCTACTTACAG  AGAAGGATATAATTCCTACG | 11929 -  12793 | 166 | 865 | 55 |
| 19 | 19F  19R | AAACAACCCAGCTCTCCCTAA  TCGATGATGTGGTCTTTGGA | 12551 -  13526 | 242 | 976 | 55 |
| 20 | 20F  20R | ACATCTGTACCCACGCCTTC  AGAGGGGTCAGGGTTCATTC | 13319 -  14287 | 207 | 969 | 58 |
| 21 | 21F  21R | GCATAATTAAACTTTACTTC  AGAATATTGAGGCGCCATTG | 14081 -  15017 | 206 | 937 | 55 |
| 22 | 22F  22R | TGAAACTTCGGCTCACTCCT  AGCTTTGGGTGCTAATGGTG | 14837 -  15997 | 180 | 1161 | 58 |
| 23 | 23F  23R | TCATTGGACAAGTAGCATCC  GAGTGGTTAATAGGGTGATAG | 15792 -  31 | 205 | 809 | 58 |
| 24 | 24F  24R | CACCATCCTCCGTGAAATCA  AGGCTAAGCGTTTTGAGCTG | 16401 -  794 | 199 | 963 | 58 |

^1^Annealing temperature
