# Supplementary material for: Mitochondrial Genome Variations in Advanced Stage Endometriosis: A Study in South Indian Population
Source: PLoS One. 2012 Jul 17;7(7):e40668. doi: 10.1371/journal.pone.0040668 (PMC3398934; doi:10.1371/journal.pone.0040668)
Supplement: Table S2 — mtDNA novel synonymous mutations observed in endometriosis patients. (DOC) [file pone.0040668.s004.doc]

**Table S2**

**mtDNA novel synonymous mutations observed in endometriosis patients^1^**

| **Gene/**  **region** | **Nucleotide position** | **Ref** | **Base change** | | | **Germline/ Somatic^2^** | **F** | **Codon & AA change** |
| --- | --- | --- | --- | --- | --- | --- | --- | --- |
|  |  |  | **Bld** | **Eut** | **Ect** |  |  |  |
| ND1 | C3486T | C | T | T | T | germ-line | 7 | P60P |
| ND1 | C3573T | C | C | C | T | somatic | 3 | L89L |
| ND2 | A4487G | A | G | G | G | germ-line | 1 | E6E |
| ND2 | C4604A | C | A | A | A | germ-line | 1 | T45T |
| ND2 | C4730T | C | T | T | T | germ-line | 1 | T87T |
| ND2 | C4799T | C | C | C | T | somatic | 1 | P110P |
| ND2 | T5082C | T | C | C | C | germ-line | 7 | L205L |
| ND2 | A5258G | A | G | G | G | germ-line | 1 | K263K |
| COI | C6959T | C | T | T | T | germ-line | 1 | G352G |
| COI | A6974G | A | G | G | G | germ-line | 1 | V357V |
| COI | C7019T | C | C | C | T | somatic | 1 | Y372Y |
| COI | C7280T | C | T | T | T | germ-line | 1 | F459F |
| COI | T7310C | T | C | C | C | germ-line | 2 | I469I |
| COI | G7382A | G | A | A | A | germ-line | 1 | E493E |
| COII | A8116G | A | A | A | G | somatic | 1 | G177G |
| ATPase 6 | C9061T | C | C | C | T | somatic | 1 | L179L |
| ATPase 6 | T9126A | T | A | A | A | germ-line | 1 | T200T |
| COIII | A9398G | A | G | G | G | germ-line | 1 | E64E |
| COIII | C9650T | C | C | C | T | somatic | 1 | H148H |
| COIII | C9773T | C | T | T | T | germ-line | 1 | S189S |
| ND3 | C10128T | C | T | T | T | germ-line | 1 | L24L |
| ND4 | C10852T | C | T | T | T | germ-line | 1 | S31S |
| ND4 | T11399C | T | T | T | C | somatic | 1 | L204L |
| ND4 | C11542T | C | C | C | T | somatic | 1 | F261F |
| ND4 | A11707G | A | A | A | G | somatic | 1 | M316M |
| ND5 | C12789T | C | T | T | T | germ-line | 1 | S151S |
| ND5 | C12867T | C | C | C | T | somatic | 1 | I177I |
| ND5 | C13125T | C | T | T | T | germ-line | 1 | F263F |
| ND5 | A13866G | A | G | G | G | germ-line | 1 | K510K |
| Cytb | C14920T | C | T | T | T | germ-line | 1 | D58D |
| Cytb | C15094T | C | T | T | T | germ-line | 5 | G116G |
| Cytb | C15224T | C | T | T | T | germ-line | 1 | L160L |

**^1^**Total number of mutatons: 32; **^2^**Germ-line mutations: 22, Somatic mutations: 10;

**Ref:** Cambridge reference sequence; **Bld:** Blood; **Eut:** Eutopic endometrium;

**Ect:** Ectopic endometrium; **F:** Frequency of mutations
