# Supplementary material for: Mitochondrial Genome Variations in Advanced Stage Endometriosis: A Study in South Indian Population
Source: PLoS One. 2012 Jul 17;7(7):e40668. doi: 10.1371/journal.pone.0040668 (PMC3398934; doi:10.1371/journal.pone.0040668)
Supplement: Table S3 — mtDNA novel non-protein coding region mutations observed in endometriosis patients. (DOC) [file pone.0040668.s005.doc]

**Table S3.**

| **Gene/**  **region** | **Nucleotide position** | **Ref** | **Base change** | | | **Eut** → **Ect pattern** | **Germ-line/ Somatic^2^** | **F** |
| --- | --- | --- | --- | --- | --- | --- | --- | --- |
|  |  |  | **Bld** | **Eut** | **Ect** |  |  |  |
| D-loop | A16T | A | T | T | T | HM → HM | germ-line | 1 |
| D-loop | A56 del | A | A | A | - | HM → HM | somatic | 1 |
| D-loop | T58A | T | A | A | A | HM → HM | germ-line | 1 |
| D-loop | 65 ins T | - | T | T | T | HM → HM | germ-line | 1 |
| D-loop | C105 del | C | C | C | - | HM → HM | somatic | 2 |
| D-loop | C113T | C | T | T | T | HM → HM | germ-line | 1 |
| D-loop | 149 ins T | - | - | - | T | HM → HM | somatic | 1 |
| D-loop | 356 ins C | - | C | C | C | HM → HM | germ-line | 1 |
| D-loop | C394T | C | C | C | T | HM → HM | somatic | 1 |
| D-loop | 401 ins C | - | C | C | C | HM → HM | germ-line | 3 |
| D-loop | 402 ins A | - | A | A | A | HM → HM | germ-line | 6 |
| D-loop | 403 ins T | - | T | T | T | HM → HM | germ-line | 15 |
| D-loop | A438G | A | A | A | G | HM → HM | somatic | 1 |
| D-loop | 524 ins A | - | A | A | A | HM → HM | germ-line | 4 |
| 12S rRNA | C775C/G | C | C | C | C/G | HM → HT | somatic | 1 |
| 12S rRNA | G811G/A | G | G | G | G/A | HM → HT | somatic | 3 |
| 12S rRNA | A1171A/G | A | A | A | A/G | HM → HT | somatic | 10 |
| 12S rRNA | A1171G | A | G | G | G | HM → HM | germ-line | 1 |
| 12S rRNA | A1240G | A | G | G | G | HM → HM | germ-line | 1 |
| 12S rRNA | A1317T | A | T | T | T | HM → HM | germ-line | 1 |
| 12S rRNA | A1383G | A | A | A | G | HM → HM | somatic | 1 |
| tRNA val | T1607C | T | C | C | C | HM → HM | germ-line | 1 |
| 16S rRNA | C1685A | C | A | A | A | HM → HM | germ-line | 1 |
| 16S rRNA | C2005T | C | C | C | T | HM → HM | somatic | 1 |
| 16S rRNA | C2414A | C | A | A | A | HM → HM | germ-line | 1 |
| 16S rRNA | G2536G/A | G | G | G | G/A | HM → HT | somatic | 30 |
| 16S rRNA | C2756T | C | T | T | T | HM → HM | germ-line | 1 |
| 16S rRNA | C2961T | C | C | C | T | HM → HM | somatic | 1 |
| 16S rRNA | 3229 ins A | - | A | A | A | HM → HM | germ-line | 1 |
| tRNA Gln | T4353C | T | C | C | C | HM → HM | germ-line | 1 |
| tRNA Cys | A5807G | A | G | G | G | HM → HM | germ-line | 1 |
| tRNA His | T12167C | T | C | C | C | HM → HM | germ-line | 1 |
| tRNA Thr | C15926T | C | C | C | T | HM → HM | somatic | 2 |
| D-loop | 16351 ins C | - | - | - | C | HM → HM | somatic | 3 |

**mtDNA novel non-protein coding region mutations observed in endometriosis patients^1^**

**^1^**Total number of mutations: 34; **^2^**Germ-line mutations: 20, Somatic mutations: 14;

**Ref:** Cambridge reference sequence; **Bld:** Blood; **Eut:** Eutopic endometrium; **Ect:** Ectopic endometrium; **HM:** Homoplasmic mutation; **HT:** Heteroplasmic mutation; **F:** Frequency of mutations
